# ‘I am on treatment since 5 months but I have not received any money’: coverage, delays and implementation challenges of ‘Direct Benefit Transfer’ for tuberculosis patients – a mixed-methods study from South India

**DOI:** 10.1080/16549716.2019.1633725

**Published:** 2019-07-22

**Authors:** Abhay Subhashrao Nirgude, Ajay M. V. Kumar, Timire Collins, Poonam Ramesh Naik, Malik Parmar, Li Tao, Kibballi Madhukeshwar Akshaya, Pracheth Raghuveer, Santosh K. Yatnatti, Navya Nagendra, Sharath B. Nagaraja, Shaira Habeena, Badarudeen MN, Ramkrishna Rao, Suresh Shastri

**Affiliations:** a Department of Community Medicine, Yenepoya Medical College, Yenepoya (Deemed To Be University), Mangaluru, India; b Department of Research, International Union Against Tuberculosis and Lung Disease (The Union), Paris, France; c Department of Research, The Union South-East Asia Office, New Delhi, India; d Center for Operations Research, International Union Against Tuberculosis and LungDisease, Harare, Zimbabwe; e Communicable Disease Section (Tuberculosis), WHO Country Office for India, New Delhi, India; f National Center for Tuberculosis Control and Prevention, China CDC, Beijing, China; g Department of Community Medicine, ESIC Medical College and PGIMSR, Bengaluru, India; h Health and Family Welfare Department, Mangaluru, India; i Karnataka State AIDS Prevention Society and State Tuberculosis Cell, Bangalore, Karnataka, India

**Keywords:** Conditional cash transfer, cash incentives, nutritional support, catastrophic expenditure, operational research, SORT IT

## Abstract

**Background**: In March 2018, the Government of India launched a direct benefit transfer (DBT) scheme to provide nutritional support for all tuberculosis (TB) patients in line with END TB strategy. Here, the money (@INR 500 [~8 USD] per month) is deposited electronically into the bank accounts of beneficiaries. To avail the benefit, patients are to be notified in NIKSHAY (web-based notification portal of India’s national TB programme) and provide bank account details. Once these details are entered into NIKSHAY, checked and approved by the TB programme officials, it is sent to the public financial management system (PFMS) portal for further processing and payment.

**Objectives**: To assess the coverage and implementation barriers of DBT among TB patients notified during April–June 2018 and residing in Dakshina Kannada, a district in South India.

**Methods**: This was a convergent mixed-methods study involving cohort analysis of patient data from NIKSHAY and thematic analysis of in-depth interviews of providers and patients.

**Results**: Of 417 patients, 208 (49.9%) received approvals for payment by PFMS and 119 (28.7%) got paid by 1 December 2018 (censor date). Reasons for not receiving DBT included (i) not having a bank account especially among migrant labourers in urban areas, (ii) refusal to avail DBT by rich patients and those with confidentiality concerns, (iii) lack of knowledge and (iv) perception that money was too little to meet the needs. The median (IQR) delay from diagnosis to payment was 101 (67–173) days. Delays were related to the complexity of processes requiring multiple layers of approval and paper-based documentation which overburdened the staff, bulk processing once-a-month and technological challenges (poor connectivity and issues related to NIKSHAY and PFMS portals).

**Conclusion**: DBT coverage was low and there were substantial delays. Implementation barriers need to be addressed urgently to improve uptake and efficiency. The TB programme has begun to take action.

## Background

Tuberculosis remains a leading infectious cause of mortality and morbidity globally, disproportionally affecting the poor, undernourished, vulnerable and marginalized populations [1,2]. This is witnessed by the low TB burden in countries with high social protection and vice-versa [3]. Evidence from several countries including India shows that TB causes catastrophic economic effects on the patients and their households [4–10]. Ensuring that no TB-affected families face catastrophic expenditure by 2020 is one of the goals of the END TB strategy of the World Health Organization (WHO) [11].

To achieve this, both universal health coverage (which addresses direct medical costs) and social protection measures (which addresses direct non-medical and indirect costs) will be required [12]. An economic modelling study indicates that a TB-specific approach focussing on TB patients might be more cost-effective than a TB-sensitive approach focussing on all people at high risk of developing TB [13].

In line with this, the National Strategic Plan (NSP) for TB Elimination in India 2017–25 envisages instituting several patient support and social protection measures in addition to providing free diagnosis and treatment services [14]. In March 2018, the Government of India launched a scheme called ‘Nikshay Poshan Yojana’ to provide nutritional support to TB patients. Under this scheme, all TB patients notified and treated as on or after 1 April 2018 are eligible to receive the benefit [15]. The benefit is either in kind (for example, a food basket or dry ration) or in cash (@ 500 INR [~USD 8] per month), which is transferred electronically to the bank accounts of the beneficiaries (direct benefit transfer, DBT). This information technology-enabled transfer is intended to bring in transparency and to prevent leakages, diversions and delays in the transfer of benefits to the rightful beneficiaries.

The Government of Karnataka, a state in South India, also began implementation of DBT from 1 April 2018. The successful implementation of this system requires that the beneficiaries have a bank account, an Aadhaar number (a 12-digit unique identification number provided to the residents of India, although this is not mandatory anymore) and are notified in an online TB notification system of India’s Revised National Tuberculosis Control Programme (RNTCP) called NIKSHAY [16].

There are concerns that some of the TB patients may not fulfil these pre-requisites, as has been seen in a previous study among pregnant women from South India [17]. Another study from Peru reported several implementation challenges, which included unwillingness of TB patients to reveal their bank account details, displeasure about insufficient incentives and dislike for home visits by health workers [18].

Several cash transfer schemes have been implemented in India in the past outside of health field for education, pension, nutrition, providing fuel subsidies and so on [19]. In the health field, this has been implemented to promote maternal and child health [20]. This is the first time a TB-specific DBT approach is being implemented in India. Measuring the coverage of this scheme and understanding the early implementation challenges will help in optimising the programme and maximize the desired effects. Assessing this will require a combination of both quantitative and qualitative research methods.

In this mixed-methods study, we aimed to determine among the TB patients notified in a district of South India from April–June 2018 (i) the proportion who were ‘approved for payment’ by the public financial management system (PFMS), (ii) the proportion who received the payment, (iii) the delays involved in cash transfer and (iv) factors associated with ‘non-approval of payment’. We also sought to understand the early implementation of barriers from the perspective of TB patients and healthcare providers.

## Methods

### Study design

This was a convergent mixed-methods study design with a quantitative (cohort study involving analysis of secondary data routinely collected by the programme) and a qualitative component (descriptive study involving in-depth interviews) [21].

### Study setting

The study was conducted in Dakshina Kannada district, a coastal district of Karnataka State in South India, which has a population of 2.1 million (2011 census). The district ranks first in the state in terms of literacy, with the highest literacy rate of 90% [22].

The district has a multi-tiered health system which includes primary, secondary and tertiary health care facilities that include two government hospitals at the district level and eight private medical college hospitals. TB services are delivered by the general health system under the leadership of a district TB officer (DTO) and the district health officer (DHO). To facilitate supervision and monitoring of the TB programme, the district is subdivided into seven tuberculosis units (TU). Each TU has a designated medical officer (MO-TU) and supervisory staff [senior treatment supervisor (STS) and senior TB laboratory supervisor (STLS)] who support the peripheral health institutions under each TU. To deliver TB services in the urban areas and private medical colleges, contractual staff are provided by the Revised National TB Control Programme (RNTCP) which includes TB health visitor (TBHV) and a laboratory technician (LT). There is a district accounts office which hosts the Public Financial Management System (PFMS) web portal and is involved in DBT.

### DBT process

**Figure 1.**
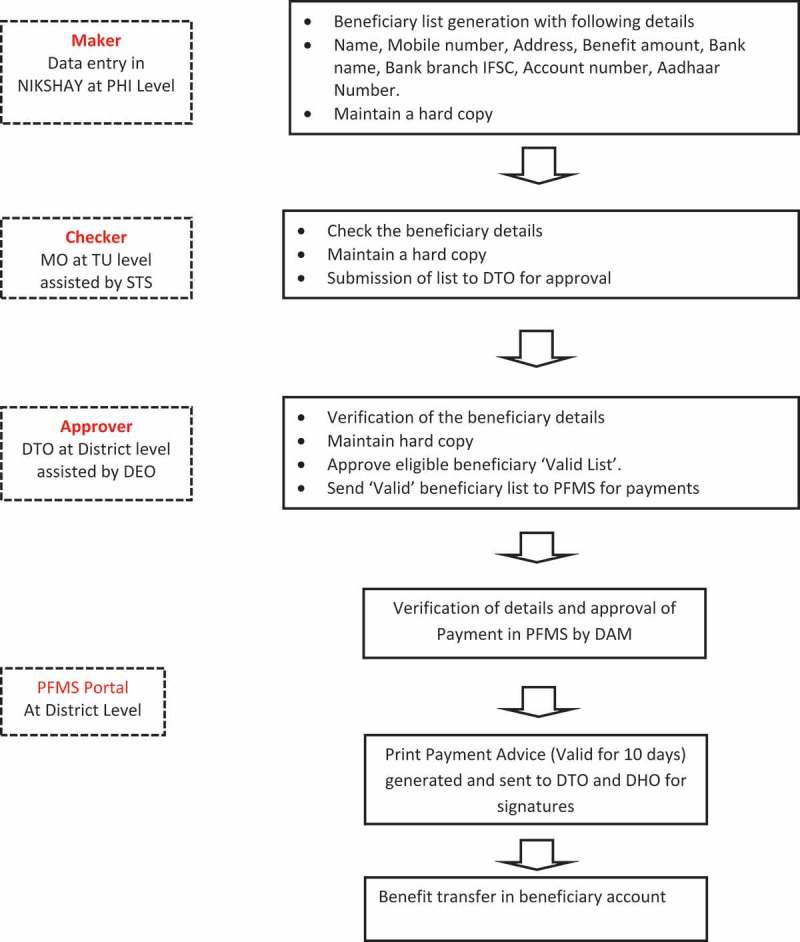
Steps in direct benefit transfer among tuberculosis patients notified in Dakshina Kannada District, Karnataka State, India, 2018. PHI = Peripheral Health Institute; PFMS = Public Financial Management System; DTO = District Tuberculosis Officer; DHO = District Health Officer; STS = Senior Treatment Supervisor; DEO = Data Entry Operator; IFSC = Indian Financial Security Code; DAM = District Accounts Manager; NIKSHAY = Online TB notification portal; Aadhaar number = unique identification number for residents of India

The process is summarized in Figure 1 and is briefly described below. Every TB patient notified on NIKSHAY (upgraded version 2.0 was introduced in September 2018) from the public as well as the private sector is eligible for receiving DBT. There are three levels of checks: the maker, the checker and the approver. The first step is to collect the patient details (name of the bank, name of the branch along with its code termed formally as Indian Financial System Code (IFSC), name of the beneficiary as it appears on the bank account, Aadhaar number wherever available) and enter into NIKSHAY. This is done by the ‘Maker’, a person identified for the purpose by the medical officer-in-charge of the Peripheral Health Institute (PHI). In urban areas and medical colleges, this is done by the TBHV. The maker then prepares the list of beneficiaries and sends it to the ‘Checker’ (MO-TU). The checker validates the details entered and sends the list to the DTO for approval. The DTO further verifies the details, removes any duplicate patient entries and approves the payment. The approved beneficiary list is then ‘pushed’ to the PFMS portal via the NIKSHAY-PFMS interface. There is a mechanism for checker and approver to validate themselves by entering a one-time password (OTP) received on their registered mobile phone number. This step is essential for sending the list to the next level.

At the district accounts office, the beneficiary details are verified again, and payment is initiated by the district accounts manager (this step is termed as ‘approval by PFMS’). Following this step, a print paper advice is generated and sent back to the DTO and DHO for signatures, which is then submitted to the bank having the RNTCP account for cash transfer.

If the patient details are not validated at any step, the respective maker or checker is notified, who is then expected to contact the patients and correct the details in NIKSHAY. During the study, this process was being done once a month with the maker, checker and approver performing their roles by 1^st^, 3^rd^ and 7^th^ of every month [23]. The same procedure is followed for every subsequent transfer to the patient. It was decided to transfer an amount of INR 1000 for every two months in order to reduce the number of transfers that needed to be done for each patient.

### Study population

#### Quantitative

All TB patients notified from April to June 2018, treated by RNTCP and residing in Dakshina Kannada district were included in the study. We excluded patients who belonged to other districts and states as they are transferred out for treatment to their respective places and are expected to receive DBT there. The programme, in its initial implementation phase, lacked clarity on DBT guidelines for patients treated in the private sector. Therefore, we excluded patients treated in the private sector.

#### Qualitative

TB patients and healthcare providers involved in the implementation of DBT were included and interviewed. We used purposive sampling to select the participants. We interviewed 10 TB patients, of whom 7 received the benefits and 3 did not. We interviewed a total of 10 healthcare providers to represent the different cadres involved in DBT implementation process. This included a laboratory technician (maker), two medical officers (checker), three STLS or STS, one TBHV of a Medical college, the data entry operator at district level, the district accountant (DTC), the district programme manager (at approver level), and the district accounts manager (PFMS). The sample size was guided by saturation of findings.

### Data collection

#### Quantitative

We extracted data variables related to the study objectives from the NIKSHAY database on 1 December 2018 (censor date). Thus, each patient's data were followed for at least 5 months. The variables included demographic and clinical characteristics, bank account number, Aadhaar number, payment approval by PFMS, payment credit along with dates of diagnosis, payment approval and payment.

#### Qualitative

In-depth interviews were conducted using an interview guide at a convenient place and time in the vernacular language or English as applicable. All the interviewers were medical doctors working as teaching faculty in a private medical college, and were trained and experienced in qualitative research methods. None of them was a part of the DBT/RNTCP implementation team. Interviews were audio-recorded after receiving written informed consent from the participants. In case participants did not consent for audio recording, notes were taken by investigators. At the end of each interview, participants were debriefed and provided an opportunity to clarify as a way of ‘member checking’. Repeat interviews were conducted as and when required to explore in-depth and explain the findings of the quantitative analysis.

### Data analysis

#### Quantitative

Data were analysed using EpiData (v2.2.2.186) and STATA (v12.1) software. Continuous data were summarized using mean and standard deviation (SD) or median and interquartile range (IQR), as applicable. Categorical data were summarized as proportions.

We chose ‘non-approval for payment’ (by PFMS) as our key outcome instead of ‘non-payment’ because once approved for payment by PFMS, it was only a matter of time before the money got transferred to the beneficiary. To assess the factors associated with ‘non-approval of payment’, we used Poisson regression and calculated adjusted relative risks and 95% confidence intervals. Since our approach was exploratory, we entered all the available variables into the regression model.

#### Qualitative

The audio recordings were transcribed within 48 h of the interview. Thematic analysis by manual coding was then carried out by four researchers (ASN, PN, KMA, SKY) independently to generate various categories or themes under the broad topics: patient-related and health system-related barriers [24,25]. The analysis was done after every interview and the findings shared among the four interviewers. This helped in identifying the emerging themes and areas that needed further probing in future interviews. This iterative process also helped in assessing the saturation of findings. The transcripts and the analysis were reviewed by other investigators (AMVK, CT) to reduce subjectivity in analysis and increase interpretive credibility. Any difference between the researchers was resolved by discussion and consensus. To ensure the confidentiality of the study participants, we have deliberately not mentioned the designation of healthcare providers in the quotes.

## Results

**Figure 2.**
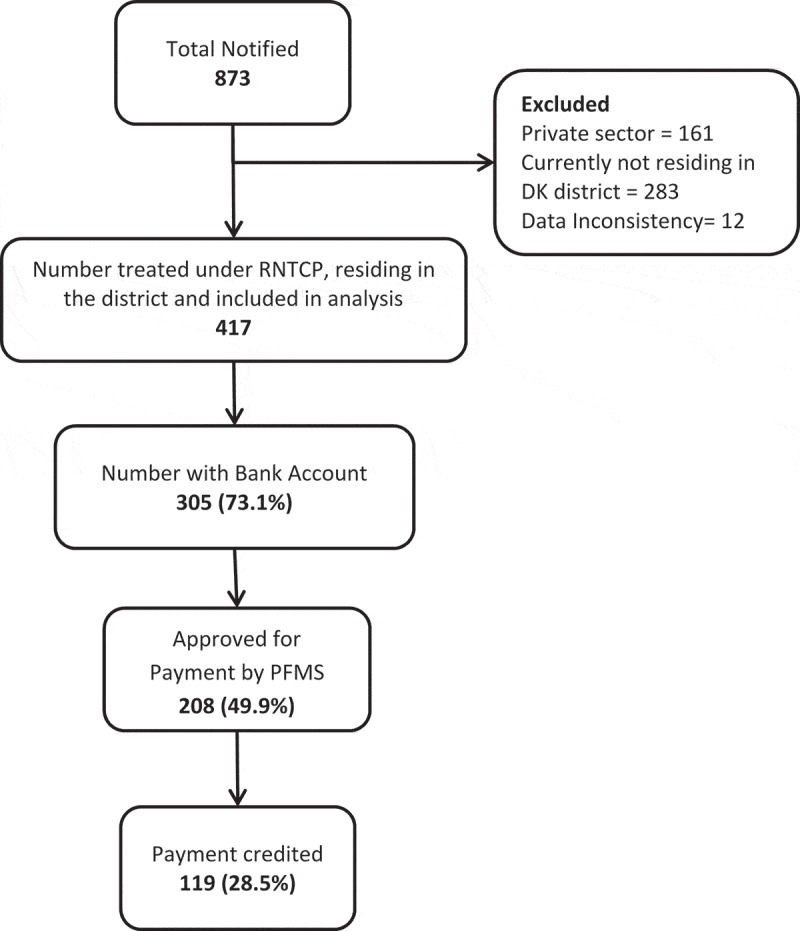
Coverage of direct benefit transfer among tuberculosis patients in Dakshina Kannada district, Karnataka state, India from April to June 2018. RNTCP = Revised National Tuberculosis Control Programme; PFMS = Public Financial Management System

Of the 873 patients notified in NIKSHAY, 417 patients fulfilled the study eligibility criteria and were included in the analysis. Of the latter, 208 (49.9%) had been approved for payment by the PFMS and of them, 119 (28.7%) were paid (Figure 2).

The demographic and clinical characteristics of the participants are shown in Table 1. The mean (SD) age was 42 (17) years and 69.3% were males. Majority (62.6%) lived in urban areas. Most (95%) had a phone number. Many patients had missing data on key clinical variables such as HIV status (46.6%), type of TB (27.3%) and disease site (27.3%). Nearly one-fourth of the patients did not have Aadhaar numbers and bank accounts.

**Table 1.** Characteristics of TB patients treated in the public sector and notified in Dakshina Kannada district, Karnataka state, India, April 2018 to June 2018 (*N* = 417).

Variable	Number	Percentage
**Age group in years**		
0–17	17	04.1
18–44	208	49.9
45–59	121	29.0
60 and above	71	17.0
**Gender**		
Female	128	30.7
Male	289	69.3
**TB unit**		
Bantwal	40	09.6
Belthangady	18	04.3
Mangaluru North	220	52.8
Mangaluru South	32	07.7
Moodabidre	25	06.0
Puttur	63	15.1
Sullia	19	04.6
**Type of TB case**		
New	276	66.2
Retreatment	27	06.5
Not recorded	114	27.3
**Disease site**		
Pulmonary	227	54.4
Extra-Pulmonary	76	18.2
Not recorded	114	27.3
**HIV status**		
Reactive	5	01.2
Non-Reactive	88	21.1
Not Recorded	324	77.7
**Diagnostic test**		
Microscopy	206	49.4
Xpert MTB/RIF	98	23.5
Other	113	27.1
**Residence**		
Rural	156	37.4
Urban	261	62.6
**Bank account**		
Yes	305	73.1
No	112	26.9
Aadhaar number*		
Yes	292	70.0
No	125	30.0

* 12-digit unique identification number provided to the residents of India.

In multivariate analysis, not having a bank account was independently associated with ‘non-approval of payment’. Although there were differences between urban and rural areas, it did not reach statistical significance (Table 2).

**Table 2.** Factors associated with non-approval of payment among tuberculosis patients in Dakshina Kannada district, Karnataka state, India, April–June 2018.

		Non-approval of payment					
Variable	Total	*N*	%	RR	95% CI	aRR	95% CI
**Age group (years)**							
0–17	17	06	35.3	1		1	
18–44	208	100	48.1	1.36	0.70–2.63	1.22	0.49–2.99
45–59	121	65	53.7	1.52	0.78–2.95	1.23	0.48–3.13
60 and above	71	38	53.5	1.51	0.76–2.99	1.14	0.44–2.98
**Gender**							
Female	128	61	47.7	1		1	
Male	289	148	51.3	0.47	0.39–0.57	0.97	0.70–1.34
**TB unit**							
Bantwal	40	11	27.5	1		1	
Belthangady	18	3	16.7	0.60	0.92–1.91	0.58	0.16–2.11
Mangaluru North	220	117	53.2	**1.93**	**1.36–3.80**	1.26	0.65–2.44
Mangaluru South	32	14	43.8	1.59	0.84–3.24	1.36	0.60–3.10
Moodabidre	25	17	68.0	**2.47**	**1.39–4.37**	1.63	0.74–3.60
Puttur	63	38	60.0	**2.19**	**1.27–3.76**	1.59	0.77–3.26
Sullia	19	09	47.4	1.72	0.86–3.43	1.40	0.55–3.57
**Type of TB**							
New	276	114	41.3	1		1	
Retreatment	27	8	29.6	0.71	0.39–1.30	0.82	0.39–1.74
Not recorded	114	87	76.3	**1.84**	**1.55–2.19**	1*	
**Disease site**							
Pulmonary	227	87	38.3	1		1	
Extra-Pulmonary	76	35	46.1	1.20	0.89–1.61	1.47	0.83–2.63
Not recorded	114	87	76.3	**1.99**	**1.63–2.41**	1.01	0.70–1.46
**HIV status**							
Reactive	5	5	100	**2.24**	**1.99–2.52**	0.96	0.37–2.48
Non-Reactive	88	53	60.2	**1.29**	**1.05–1.58**	0.88	0.62–1.25
NotRecorded	324	151	46.6	1		1	
**Diagnostic test**							
Microscopy	206	100	48.5	1		1	
Xpert MTB/RIF	98	62	63.3	**1.30**	**1.06–1.60**	0.98	0.68–1.43
Other	113	47	41.6	0.85	0.66–1.11	0.81	0.47–1.39
**Residence**							
Rural	156	60	45.6	1		1	
Urban	261	149	64.7	**1.48**	**1.18–1.85**	1.30	0.94–1.80
**Aadhaar number**							
Yes	305	107	36.6	1		1	
No	112	102	81.6	**2.22**	**1.87–2.64**	0.99	0.67–1.47
**Bank account**							
Yes	305	97	31.8	1		1	
No	112	112	100	**3.29**	**2.79–3.87**	**3.07**	**2.01–4.67**

*Omitted due to collinearity with the variable disease site.

TB = tuberculosis; HIV = human immunodeficiency virus; Aadhaar number = 12-digit unique identification number provided to citizens of India; RR = unadjusted relative risk; aRR = adjusted relative risk; CI = confidence intervals;

RR and 95% CI in bold indicate variables found to be statistically significant (*p* value <0.05)

### Delays

Overall, the median (IQR) delay from diagnosis to payment was 101 (67–173) days – contributed by the delay from diagnosis to ‘approval for payment’ which was 74 (50–122) days and the delay from approval to payment which was 10 (9–16) days. The delay in payment was significantly greater among patients from rural areas (median of 126 days) compared to urban areas (85 days) (p < 0.001).

### Qualitative

**Figure 3.**
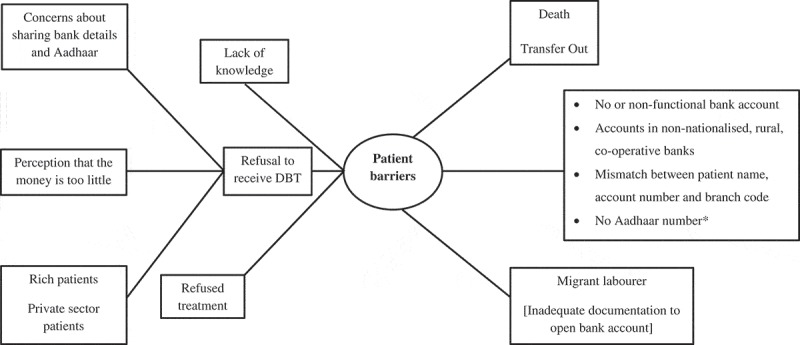
Non-hierarchical thematic map showing patient-related barriers in the implementation of DBT for TB patients notified in Dakshina Kannada district, Karnataka state, India, April-2018 to June-2018. * 12-digit unique identification number provided to residents of India; DBT = Direct Benefit Transfer; TB = Tuberculosis

**Figure 4.**
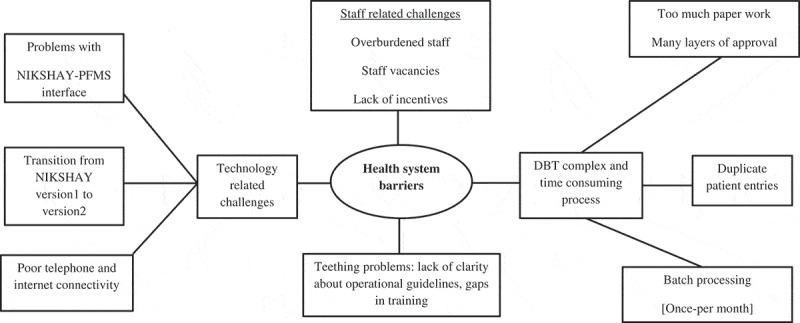
Non-hierarchical thematic map showing health system-related barriers in the implementation of DBT for TB patients notified in Dakshina Kannada district, Karnataka state, India, April-2018 to June-2018. DBT = Direct Benefit Transfer; TB = Tuberculosis; NIKSHAY = online TB notification portal; PFMS = Public Financial Management System

The DBT scheme was welcomed and appreciated by both patients and healthcare providers as a noble initiative aimed at providing nutritional support to all TB patients, who are mostly *poor, too sick to work* and lack social support. DBT was also viewed as a measure preventing misuse and pilferage of funds. However, several implementation barriers were noted. We describe these barriers below under two broad organizing themes – patient barriers and health system barriers (Figures 3 and 4). The patient-related barriers summarized here reflect the perspectives of the patients as well as the healthcare providers.

### Patient-related barriers

#### No bank account

Not having a bank account was a key challenge noted, especially among the migrant labourers in the district. In some patients, healthcare providers explored transferring the money to one of the family member’s bank accounts. If this was not possible, efforts were made with the assistance of local non-governmental organizations, to help patients open a bank account. But, such efforts were often unsuccessful because patients lacked essential documents (like proof of address) to open an account. Some banks insisted on depositing a minimum amount at the time of opening the account, which was often not possible for poor patients who were destitute and admitted in the TB sanatorium.

So overall the people admitted in TB sanatorium who are destitute are not getting the benefit … I am helpless. (Health care provider)

Some patients had their accounts in rural, co-operative banks, with challenges in electronic fund transfer, thus leading to delays. In some patients, bank transfer was unsuccessful because of non-functional account or mismatches between account number, account holder’s name and the branch code. Re-initiating transfers after correcting these added to the delay. Rarely, a few patients had multiple bank accounts and this unnecessarily led to some confusion as the money always got deposited to Aadhaar-linked bank account, despite them giving another account at the time of registration.

#### No aadhaar

Lack of Aadhaar card was one of the early implementation challenges, which was a mandatory requirement initially, but was made optional later.

#### Refusal

Some patients who were financially sound and most often, receiving treatment from the private sector refused to receive DBT. Some patients had confidentiality concerns about sharing their bank details as they feared this would reveal their TB status to people outside the health sector.

I don’t want any benefit from Government. I earn 50,000 per month and I work in XXXX. I don’t need this 500 rupees. Government simply tells that they will give money but it will never be given to patients.

#### Money inadequate

Many patients felt that the money given under DBT was not sufficient to meet their needs. This feeling was echoed by the providers too, who felt that the money should be doubled.

Even for three days around 200 rupees required then how 500 rupees will be sufficient for one month?(Patient)

#### Not aware

While most patients interviewed were aware of DBT, one was unaware despite being on treatment for six months.

I am on treatment since 6 months and I have not submitted any bank details or Aadhaar card details to anyone and unaware of DBT scheme. (Patient)

### Health system-related barriers

#### Teething problems

This included a lack of clarity about operational guidelines of implementation including roles and responsibilities. Trainings came late and affected the smooth take-off of the DBT programme.

We were not sure in the beginning of the DBT program “whether Aadhaar is mandatory or not? Who will get the money? Who will do the job of Maker in absence of data entry operator?” (Health care provider)

#### DBT is complex

The key challenge noted by the providers was that DBT was a complex and time-consuming process with many layers of approval and involved too much paperwork.

*The process is too lengthy … the file movement, which starts from the DEO* [Data entry operator] *to DPC* [District Programme coordinator], *from DPC to DPM*[District Programme Manager], *from DPM to sir* [DTO], *from sir to PFMS and then the sheet from PFMS and to DAM* [District Accounts Manager], *from DAM again back to sir … … It should be direct transfer according to my opinion. (Health care provider)*

The programme staff pointed out that such a complex procedure caused the delays. Sometimes, the delay was so long that the patients did not receive the money until most of the treatment was over.

I am on treatment since 5 months but I have not received any money. (Patient)

The additional paper-based documentation required at every level was viewed as an extra burden to the existing workload brought by the DBT scheme. For example, even after approval by PFMS, a paper print advice (valid for 10 days) was to be sent back to the DTO and DHO for signatures and physical submission to the bank before payment. Any delay beyond 10 days meant that the process had to be repeated.

#### Staff challenges

Since there are no dedicated data entry operators, it was a challenge to identify another staff member (like laboratory technician or nurse or pharmacist) to take on the role of the ‘maker’ at the PHI level, without any additional incentives. This posed an additional burden among them, especially in PHIs with staff vacancies. In PHIs where it was not possible to identify a maker, the burden fell on the TB programme staff.

#### Technology-related challenges

Technological challenges were reported to compound the complexity of the process. First, there was a problem with the interface between NIKSHAY and PFMS, which was not always automated, as expected. Second, poor phone connectivity (failure to receive OTP), slow internet and intermittent electricity supply meant that the staff had to work for long hours to make entries and prepare beneficiary lists in NIKSHAY. Third, there were problems associated with the transition from NIKSHAY 1 to NIKSHAY 2.0, though many hoped that NIKSHAY 2 will address most of the challenges and ease the process.

#### Bulk processing

Another factor causing the delay was related to the frequency of processing the payments, which occurred once-a-month and not in real-time. Batch processing made the beneficiary list ‘*bulkier*’ which was believed to be the reason behind multiple errors including duplicate entries, undue pressure on the district staff to validate the records and inability to ‘*push the huge list*’ from NIKSHAY to PFMS. All these delayed the direct benefit (Cash) transfers.

As everything comes in bulk and additionally paper-based approval also required there will be delay in sending the details to PFMS at DTO level. (Health care provider)

### Duplication

Duplicate patient records (meaning the same patient getting notified twice in NIKSHAY) were reported to be another challenge leading occasionally to double transfer of money or delays.

At PHI and TU level beneficiary will get added twice. This will delay the approval at DTO level. As this needs verification from PHI and TU about which entry is correct. (Health care provider)

## Discussion

‘Nikshay Poshan Yojana’ in India is probably the largest direct benefit transfer programme ever-implemented globally among TB patients under programmatic conditions. Our study is the first one to assess the coverage and implementation challenges of this mammoth initiative. We discuss the key findings below.

We found that nearly half of the TB patients were not approved for payment. There are no data from a peer-reviewed publication for comparison. But, a newspaper report in December 2018 mentioned that about 25% of TB patients in Amritsar district (North India) received DBT [26]. Another report of the Global Fund oversight committee mentioned that only 6.8% of eligible TB patients in Kamrup district (North-eastern India) received DBT [27].

There were several reasons for non-approval of DBT services. The key reason was ‘not having a bank account’, in which case DBT was not possible. Efforts to open an account were often unsuccessful, especially among migrant labourers working in urban areas and very sick patients admitted in TB sanatorium. This means that the most needy patients do not get the benefit of DBT or get it too late to be of any use [28]. Some patients refused DBT because they either had confidentiality concerns about sharing the bank details or were well-off economically and did not need it. These explain why a greater proportion of patients in urban areas did not avail DBT services.

Many patients, especially those with missing details on key clinical variables, might have been lost to follow-up (LFU) or died before starting treatment and hence, their bank account details were not documented [29]. Lack of awareness about DBT among TB patients might be another reason for non-payment.

Even among patients with a bank account, about one-third did not receive DBT services. Possible reasons include wrong or non-functional bank account number and mismatches in IFSC code, account number and beneficiary name. Given the complexities of the DBT process with multiple layers of approval and the need for paper-based documentation at every level, many such patients may still be designated ‘under process’. These might also explain the long delays in cash transfer.

Other reasons for delay include (i) technology-related challenges such as slow internet and server (ii) the practice of bulk processing once-a-month instead of real-time (iii) staff vacancies at some PHIs (iv) training gaps (v) overburdened RNTCP staff and (iv) lack of clarity about operational guidelines in the initial stages of implementation. Interestingly, TB patients in rural areas had longer delays than urban areas. This might be because patients in rural areas are more likely to have bank accounts in small cooperative, non-nationalized banks, which might take a longer time for processing.

Another notable finding was that the amount provided was perceived by both patients and healthcare providers to be inadequate to meet its intended purpose of nutritional support. This is consistent with the findings from a Peruvian study [18].

DBT is not new in India. It has been used in the past to provide pension payments, cooking fuel subsidies, scholarships, employment guarantee schemes and maternity benefit schemes. Several constraints and challenges were reported in implementation of these schemes which include policy dilemmas related to the time frame of implementation, amount of cash benefit, budgetary requirement and burden on the existing human resource [19].

In India under Janani Suraksha Yojana, the eligible pregnant women (below poverty Line) receive cash benefit for institutional delivery. In a study from South India, only half of the eligible mothers received cash benefits, and the mean time to receive the benefit was 96 days (IQR 60–120 days). Non-submission of application by beneficiary and rejection of application due to lack of a bank account and Aadhaar number were the key reasons for not receiving the cash benefits [17]. Another qualitative study done in three states of India about conditional cash transfer to improve institutional deliveries reported several barriers in implementation. This includes poor quality of health care, inadequate resources, poor transport facility and attitude of the healthcare provider [30].

Globally, there have been several studies assessing the effectiveness of cash interventions on outcomes among TB patients. A systematic review synthesizing this evidence concluded that cash interventions in low- and middle-income countries resulted in positive clinical outcomes among TB patients [31]. Cash interventions varied in the form of direct bank transfer of cash, cash in hands and non-cash in the form of food vouchers. However, authors from a study in Peru reported poor acceptability and accessibility for food vouchers and justified cash transfer directly into the accounts of the TB patients as the best way to achieve the intended outcome [18].

### Strengths

First, our study was conducted under programmatic conditions, and the results reflect the field realities. Second, using a mixed-methods design helped in a comprehensive assessment of the issue. Most of the studies on conditional cash transfers among TB patients in the past have tended to focus on outcomes rather than implementation challenges [32]. Our study addresses this knowledge gap here. Since the investigators involved in the qualitative component were not a part of the DBT/RNTCP programme implementation team, it helped in ensuring objectivity in analysis and interpretation. Additionally, we had a large sample and we included all eligible patients (without sampling) in our study, thus ensuring internal validity. We adhered to Strengthening the Reporting of Observational Studies in Epidemiology (STROBE) and ‘Consolidated Criteria for Reporting Qualitative Research’ (COREQ) guidelines for reporting quantitative and qualitative components, respectively, [33,34].

### Limitations

Since the study was conducted only in one district, the findings have limited generalizability beyond the district. We believe the situation in other districts in Karnataka state with lower literacy rates and financial inclusion might be even worse [35]. We identified gaps in recording of key variables in the NIKSHAY patient database. Although it did not impact our results in a major way, this needs attention.

### Future research

We did not include patients who belonged to other districts and those treated in the private sector in our study. We also did not study the impact of DBT on reducing catastrophic health expenditure and on improving nutritional status, treatment adherence and treatment outcomes. We also did not explore if the patients used the money for nutritional support or other purposes. These may be studied in the future research.

### Programme implications

First, urgent attention should be given to very sick patients who do not have a bank account or adequate documentation to open one in time. Since these are the neediest patients, consideration should be given to pay by alternative means in exceptional cases. Second, there is a need to simplify the processes involved in DBT which includes doing away with additional paper-based documentation at every level. Third, RNTCP needs to process DBT in real-time rather than once-a-month. Fourth, there should be in-built checks within NIKSHAY to validate the accuracy of bank account number and IFSC code, so that delays due to this may be prevented. Fifth, urgent measures should be taken to resolve the NIKSHAY-PFMS interface-related challenges. Sixth, measures must be taken to improve the awareness of DBT scheme among both the patients and the healthcare providers by regular awareness campaigns and trainings. Awareness campaigns must also aim to allay fears related to confidentiality among TB patients. Seventh, the reasons for non-receipt and refusal of DBT need to be routinely documented. This will help in instituting course corrective measures. Finally, DBT should be monitored and reviewed on priority at district, state and national level review meetings. User-friendly dashboards on NIKSHAY could be a possible way for enabling real-time monitoring.

### Actions taken by RNTCP

To RNTCP’s credit, some steps aimed at easing DBT processes have already begun. For example, Aadhaar number is no more considered mandatory for DBT. The requirement for a ‘one-time password’ at maker and checker level has been removed. There is a new version of NIKSHAY which is envisaged to have inbuilt checks for validating patient details and a seamless interface with PFMS and dashboard-based real-time monitoring. We hope these would address many of the barriers identified in this study and recommend repeating the study after 6–12 months to see if these measures have had an effect on DBT coverage.

## Conclusion

In this first operational research on DBT among TB patients, we found that the coverage was low and there were significant delays in receiving the benefits. The coverage was poorer in urban areas, while delays were greater in rural areas. Key implementation barriers included ‘not having a bank account’, refusal to receive DBT, complexities in the DBT process with the requirement of multiple approvals, bulk processing and technology-related challenges. The barriers seemed to disproportionately affect the neediest patients. There is an urgent need to address these barriers to enhance the uptake and efficiency in DBT utilization. RNTCP has begun addressing some of the barriers.

## Paper context

In 2018, the Government of India launched the largest direct benefit (money) transfer programme ever implemented in the world covering millions of TB patients in the country. Unlike most previous studies which focussed on clinical outcomes, we focused on assessing coverage, which was only ~50%, and there were long delays in cash transfer. We identified several implementation barriers which will guide the programme managers to take corrective measures.
